# Vitamin D Mitigates Hyperglycemia-Induced Cognition Decline in *Danio rerio* (Zebrafish) through the Activation of Antioxidant Mechanisms

**DOI:** 10.3390/antiox11112114

**Published:** 2022-10-27

**Authors:** Chinnappa A. Uthaiah, Nandini C. Devaru, Nandini H. Shivakumar, Rajalakshmi R, SubbaRao V. Madhunapantula

**Affiliations:** 1 Department of Biochemistry (A DST-FIST Supported Department), JSS Medical College; JSS Academy of Higher Education & Research, Mysuru 570015, India; 2 Department of Pharmacology, JSS College of Pharmacy; JSS Academy of Higher Education & Research, Mysuru 570015, India; 3 Department of Physiology, JSS Medical College, JSS Academy of Higher Education & Research, Mysuru 570015, India

**Keywords:** vitamin D, cognition impairment, diabetes, Nrf2, oxidative stress, zebrafish, sulforaphane

## Abstract

Hyperglycemia contributes to the development of cognition impairment and related disorders, induces oxidative stress in neuronal cells; thereby, impairs normal signaling mechanisms involved in cognition processes. Studies have shown a significant decrease in the vitamin D in individuals with hyperglycemia and cognition impairment. But whether supplementing vitamin D has any beneficiary impact on mitigating hyperglycemia-induced cognition impairment is unknown. We have first tested the impact of hyperglycemia on the induction of cognition deficiency in a zebrafish model. Next, the molecular mechanisms related to oxidative stress, which are deregulated in hyperglycemic zebrafish brains, have been explored. Subsequently, the impact of supplementing the water with vitamin D and a known activator of nuclear factor erythroid-2 related factor 2 (Nrf2) i.e., sulforaphane (SFN) on learning and memory functions were assessed. We showed a significant increase in the oxidative stress in the brain tissue of zebrafish residing in hyperglycemic water (111 mM glucose). Addition of vitamin D and SFN increased Nrf2, but differentially modulated its target genes (NQO1, SOD, GPx etc) activity in zebrafish and neuronal cell lines thereby improved the hyperglycemia-induced decline of cognition impairment. Mechanistically, vitamin D binds to the Keap1 protein; thereby, interfering with its binding to Nrf2, which leads to the activation of antioxidant mechanisms in the cells. In summary, reducing the oxidative stress through vitamin D treatment is a possible option for controlling the cognition impairment in diabetic population, but studies testing this possibility in clinical trials are currently needed.

## 1. Introduction

Diabetes mellitus (DM) is one of the major health concerns worldwide. Type 2 Diabetes (T2D) is a metabolic disorder recognized by decreased insulin sensitivity and elevated blood glucose [1]. DM causes malfunctioning of multiple organs among which the damage caused to brain (neuropathy), eyes (retinopathy) and kidneys (nephropathy) is very prominent and widely reported [2]. According to the International Diabetes Federation (IDF), globally, the number of cases due to diabetes are predicted to increase from 537 million in 2021 to 643 million by 2030 [1].

Diabetic complications continue to progress uninterrupted even after achieving a good control over blood glucose. This phenomenon, which is known as metabolic memory or the legacy effect [3], is an independent risk factor for the development of cognitive impairment in humans [4]. Diabetes-related cognitive dysfunction is a consequence of changes within the central nervous system (CNS) [5] and is characterized by rapid or progressive destruction in energy metabolism, as well as learning and memory [5]. Furthermore, studies have demonstrated decreased utilization of cerebral glucose in conditions, such as memory dysfunction [6]. Adding to this, impaired insulin receptor signal-transduction is reported in the hypothalamus and hippocampus regions of the brain [7]. The impaired insulin signaling is associated with an increase in oxidative stress, mitochondrial- and endothelial dysfunction in neurons [8]. In addition, DM is known to alter neurotransmission systems. For example, in diabetic rats, studies have reported an increase in acethycholinesterase (AChE) mRNA and a reduction in the mRNA level of cholinergic receptors M1 and M3 [9]. Furthermore, low stimulation of cholinergic receptors leading to a progressive worsening of cognition and neurological dysfunction is also observed in diabetic patients.

The zebrafish (*Danio rerio*) is one of the preferred species for studying developmental biology [10]. Zebrafish have also been used in the neurobehavioral research due to its small size (4 cm long till adult stage), high fecundity, prolific nature, less cost, easy maintenance, rapid development and transparent embryo stage throughout its development. Moreover, the neurochemical properties and basic layout of the brain are similar to that of higher-order vertebrates, including mammals [10]. Because of these characteristics, key genetic information has also been extracted along with the development of a large array of genetic tools [10], thus making the research in the field of neurobehavioral science much easier. Zebrafish have also been used to mimic DM. Studies have shown that Type-1 DM can be modeled in zebrafish by injecting streptozotocin (STZ) [11]. Symptoms of Type-2 DM that include protein glycation, persistent hyperglycemia and impaired insulin response can be induced by immersing zebrafish in a solution of glucose [12].

Vitamin D is a neurosteroid hormone that regulates neurotransmitters and neurotrophins in various animal species [13]. It has anti-inflammatory, antioxidant, and neuroprotective properties [14]. Vitamin D increases neurotrophic factors such as nerve growth factor (NGF), which further promotes brain health and prevents the accumulation of amyloid plaques while promoting the clearance of existing amyloid deposits [15]. Emerging evidence suggests its role in the reduction in Alzheimer’s disease by mitigating the expression of amyloid-beta and phosphorylated tau proteins [15]. Many studies have supported the hypothesis that vitamin D deficiency leads to attention deficiency, behavioral abnormalities, and cognitive impairment. Cross-sectional studies have reported that vitamin D levels are significantly low in individuals with Alzheimer’s disease and cognitive impairment compared with healthy adults [16]. Longitudinal studies and meta-analysis reports have also exhibited an association of low vitamin D with cognitive impairment and Alzheimer’s disease. Despite such evidence, the absolute requirement of vitamin D for mitigating cognition abnormalities cannot be sufficiently answered due to lack of proof-of-principle studies and intervention data [17]. For instance, a set of interventional studies yielded mixed results, which raised concerns about the role of vitamin D supplementation in the prevention and treatment of cognitive impairment and dementia [18]. Moreover, the data from randomized controlled trials suffers from smaller sample size, lack of consensus over the dose, and age of initiation of vitamin D supplements to prevent cognitive impairment [19]. Therefore, there is an immediate requirement to assess the benefits of vitamin D supplementation in the prevention and treatment of cognitive impairment.

Vitamin D deficiency has been shown to impair insulin synthesis and secretion in humans and in animal models of diabetes, suggesting a role in the development of type 2 diabetes [20]. Furthermore, epidemiological studies have shown that deficiency of vitamin D in early stages of life might lead to the onset of type 1 diabetes [21]. Additional studies have demonstrated that the risk of developing diabetes and cognitive dysfunction increases with vitamin D deficiency [22]. Mechanistically, vitamin D promotes insulin synthesis and secretion while reducing insulin-resistance [23]. In addition, vitamin D enhances antioxidant systems by upregulating Nrf2 and its transcriptional targets that include superoxide dismutase (SOD), glutathione peroxidase (GPX), NADP-quinone oxidoreductase-1 (NQO1), catalase (CAT) [24]. Even though these studies have explained the possible mechanisms-of-action of vitamin D, queries pertaining to its direct targets and its interaction with cellular proteins still remain unanswered. Further studies are warranted to address these questions.

Hence, in the current study, we have evaluated the effect of vitamin D on diabetes-induced cognitive decline in the zebrafish by assessing the behavioral parameters using “T” maze. In addition, we have modeled whether vitamin D can effectively bind to the master regulator of oxidative stress, i.e., Nrf2 protein, by using in silico approaches and assessed the effect of vitamin D on antioxidant genes expression and activity. Results of these investigations have provided strong evidence, which demonstrated that vitamin D binds to Nrf2 and thereby regulate the activity of antioxidant genes SOD, GPX and NQO1 in zebra fish growing in high-glucose solution.

## 2. Materials and Methods

Maintenance of zebrafish: Wild type adult zebrafish (*Danio rerio*) of 3 to 4 months old (both sexes) measuring nearly 3–4 cm were purchased from Marine aquariums, Mysuru, KA, India. The animals were habituated in the experimental room for 7 days under controlled conditions that include (a) housing 20 fishes/group in 3 L thermostated tank maintaining the temperature between 27 °C to 30 °C. The thermostated tank has a water circulation system with a maximum cycling capacity of 400 L/hour with a lower noise ratio (25–30 dB); (b) water pH between 6.8 to 7.0 and light intensity of 250 lx (lux); and (c) Fourteen hours light (8 a.m. to 10 p.m.) and 10 h dark (10 p.m. to 8 a.m.) cycle. Animals were fed two times a day with commercially available micro pellets containing 43% crude protein, 7% each of crude fat and fiber (Kyorin, Shirogane-machi, Himeji, Japan). All protocols were scrutinized and approved by the Institutional Animal Ethics Committee (IAEC) of JSS Academy of Higher Education & Research (JSS AHER, IAEC/JSSCPM/350/2019).

**Figure 1:**
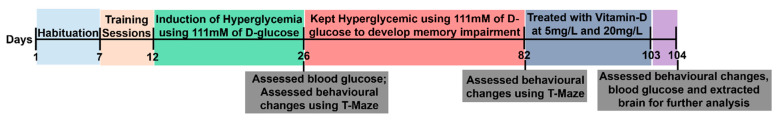
Schematic representation of the experimental protocol used in this study.

Experimental Design: Adult zebra fishes of both sexes (*n* = 15 in Group 1 and *n* = 20 in Groups 2 to 5) were used in the present study to determine the effect of vitamin-D on decreasing the severity of cognition impairment under the hyperglycemic conditions. Group 1 (*n* = 15): No hyperglycemia control; Group 2 (*n* = 20): Hyperglycemia control; Group 3 (*n* = 20): Hyperglycemic zebra fish treated with 5 mg/L vitamin D; Group 4 (*n* = 20): Hyperglycemic zebra fish treated with 20 mg/L vitamin D; Group 5 (*n* = 20): Hyperglycemic zebra fish treated with 4.4 mg/L Sulforaphane (a known activator of Nrf2 [25]). Experimentally, the fishes were habituated for 7 days (Day1 to 7), trained for 5 days (Day 8 to 12), hyperglycemia was induced by using 111 mM glucose for 14 days (Day 13 to 26). After this, the zebra fish were monitored regularly for signs of cognitive impairment development (till day 82, i.e., from Day 26 to 82). At this point, the treatment agent’s vitamin D and SFN were added, and the treatment continued for 21 days (i.e., from day 83 till day 103). On day 104 the fish were euthanized. Scheme of the experimental protocol was shown in Figure 1.

### 2.1. Assessment of Learning and Memory

Assessment of learning skills and memory by T-maze: Learning skills and memory assessment were performed using T-maze according to Kundap et al., and Paudel et al., with minor modifications [24,26]. For detailed protocol, please refer Supplementary Materials Methods section and Supplemental Figure S1a.

Training sessions: The training sessions were performed according to Bishir M et al. by measuring latency along with time spent in the deeper square chamber [27]. For additional details, please refer Supplementary Materials Methods section.

Test sessions: Once the hyperglycemia was confirmed, animals were tested for memory at 14th day (26th day from day one) and 56th day (83rd day from day one) after induction and finally at 105th day (i.e., post-treatment assessment before sacrificing the fish) by placing the fishes in the long arm. Latency time and the time spent in the deeper square compartment were recorded.

Induction of hyperglycemia and determination of blood glucose levels: Induction of hyperglycemia was carried out according to Capiotti KM et al. [11]. To determine the blood glucose levels in the animals, the procedure was followed according to Gleeson M et al. [28]. (Reference scale: Fasting blood glucose for normal fish: 50–60 mg/dL & hyperglycemic fish >110 mg/dL) [29]. Additional information about the detailed methodology can be found in Supplementary Materials Methods section.

### 2.2. Treatment with Vitamin-D and Sulforaphane

Acute toxicity assessment of vitamin-D: Acute toxicity assessment was conducted as per the Organization for Economic Cooperation and Development (OECD) guideline 203 [30] (Supporting Methods section). Sulforaphane (4.4 mg/L) was used as a standard control [31].

Gene expression analysis by quantitative real time RT-PCR (RT-qPCR): The expression of Nrf2, NQO1, Brain-Derived Neurotrophic Factor (BDNF), GPx and SOD in the whole brain tissue was analyzed by quantitative real time PCR (RT-qPCR) as detailed in Supporting Methods section. The specific primer sequences are provided in (Table 1). The melt curve was obtained, and the relative expression was quantitated using the 2^−∆∆Ct^ method [32]. The values were expressed as the relative fold change normalized to internal control β-actin.

Glutathione Peroxidase Activity (GPx) Assay: Glutathione peroxidase activity was determined in the whole brain homogenates by using commercially available high throughput glutathione peroxidase assay kit from Trevigen (catalogue no:7512-100-K). Additional information can be accessed at Supporting Methods section [33].

Superoxide Dismutase Activity (SOD) Assay: Superoxide dismutase activity was determined by using commercially available high throughput assay kit from R&D systems (Catalogue no: 7501-500-K) (Please refer to the Supporting Methods section for more information) [34].

NAD(P)H Quinone Oxidoreductase 1 (NQO1) Activity Assay: NQO1 activity assay in the whole brain homogenate was performed according to the Prochaska et al., with minor modifications as detailed in Supporting Methods section [35].

Estimation of Total Glutathione (GSH): Total GSH level in the whole brain homogenate was performed according to Rahman I. et al., (Please refer Supporting Methods section) [36].

Estimation of Acetylcholinesterase (AChE) in the Zebrafish brain: Zebrafish brains were homogenized on ice in 50 mM Tris–HCl, pH 8.0, in a glass-Teflon homogenizer. Acetylcholinesterase activity was measured as described previously. Please refer Supporting Methods section [37].

Lipid Peroxidation Assay: Lipid peroxidation was determined by estimating the thiobarbituric acid reactive substances (TBARS) as explained in the Supporting Methods section [38].

### 2.3. Molecular Docking of Vitamin D3 and Known Activators of Nrf2

In this study, Cholecalciferol (Vitamin D3) and known activators of Nrf2 viz., Sulforaphane (SFN), Pterostilbene (PTS) and Bardoxolone (CDDO) were docked into Keap-1 protein to assess and compare the binding strengths of these molecules to Keap-1 protein. We hypothesize that molecules that complex strongly with Keap1 inhibits its binding to Nrf2 thereby enhance antioxidant signaling cascades in the cells. Please refer Supporting Methods section.

Statistical Analysis: All the data were represented as Mean ± SEM in each group (*n* = 9). For the comparison between two groups student *t*-test was performed with two-tail analysis with 95% confidence interval. For the multiple group comparison, data were analyzed by one-way or two-way analysis of variance (ANOVA) followed by Tukey’s posthoc test, in which treatment group were compared with disease control and normal. The *p*-value < 0.05 considered as significant difference. Statistical analysis and graphs were plotted using GraphPad Prism software Version-5.0.

## 3. Results

**Figure 2:**
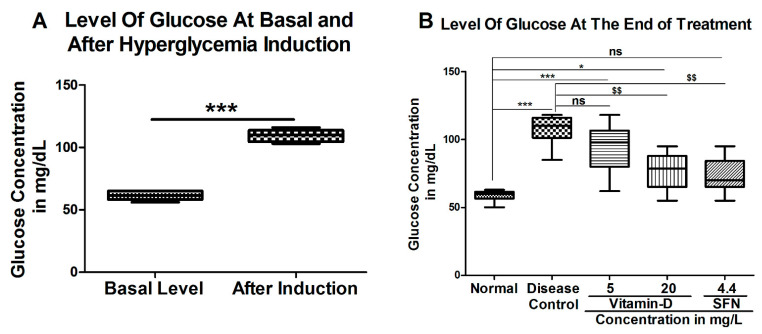
Vitamin D and SFN Reduced Blood Glucose in Diabetic Zebrafish. (**A**) Zebrafish developed hyperglycemia when grown in 111 mM glucose containing water. Data expressed as mean (*n* = 6) ± SEM. Data were analyzed by one-way ANOVA, followed by Tukey’s posthoc test. In (**A**) “***” represents a significant difference in glucose level between the basal- (before glucose treatment) and post-induction (after glucose treatment) zebrafish with *p* < 0.0001. (**B**) High dose vitamin D and SFN reduced the blood glucose in hyperglycemic zebrafish. “***, *” Represents a significant difference between the normal control group and the other groups. *p* < 0.0001 & <0.05 respectively and “$$” represents a significant difference between disease control with vitamin-D and SFN treated groups *p* < 0.001, “ns” represents ‘no significant difference.

Vitamin D reduced blood glucose level in hyperglycemic zebrafish: To determine the impact of vitamin D on reducing the blood glucose level in hyperglycemic zebrafish, a well-established model as detailed in materials and methods was used and the data was represented in Figure 2. Experimentally, sterile water containing 111 mM glucose was used to induce hyperglycemia in Zebra fish. The immersion of zebra fish in 111 mM glucose (Figure 2A) elevated blood glucose from 61.33 ± 1.48 mg/dL to 109.5 ± 2.04 mg/dL. Treatment of hyperglycemic fish with 20 mg/L of vitamin D and positive control 4.4 mg/L sulforaphane significantly decreased the blood glucose level (Figure 2B). For instance, 20 mg/L vitamin D reduced blood glucose to 77.25 ± 3.75 mg/dL. Similarly, the positive control SFN has reduced the blood glucose to 73.17 ± 3.41 mg/dL. But there was no significant decrease in blood glucose when treated with 5 mg/L vitamin D (93.38 ± 4.89). At the end of treatment, even though vitamin D (20 mg/L) significantly lowered the glucose compared with diabetic fish, the level has not reached to the level that was found in normal control fish (58.56 ± 1.33) (Figure 2B).

**Figure 3:**
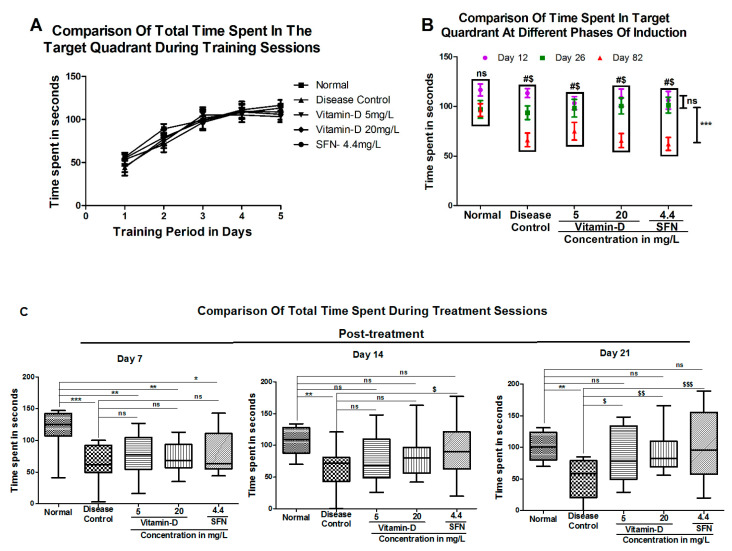
Vitamin D and SFN Treated Zebrafish Exhibited Similar Favorable Chamber Residing Time as That of Control Fish: (**A**) A 5-days training increased the residing time of zebrafish in favorable chamber. Time spent (in seconds) by zebrafish (*n* = 15) in the deep square chamber has increased gradually during the 5-days of training session. No difference in the favorable chamber residing time was observed among the experimental groups (*p* > 0.05 by One-Way ANOVA). (**B**) Hyperglycemic zebrafish spent much lesser time in the favorable chamber compared with normal zebrafish. A significant decrease in the time spent was observed in zebrafish with hyperglycemia compared with the one with no hyperglycemia, in particular at day 82 (Red triangles; *p* < 0.05 by One-Way ANOVA, Tukey’s post-hoc test). The difference is reflected in all the experimental groups. The data represents mean (*n* = 15) ± SEM. “***” Represents significance between day 12 and day 82 with *p* < 0.001, “#” Represents significance in time spent between day 26 and day 82 at *p*-value < 0.05, “$” Represents significance in time spent compared with normal control group at day 82 with *p*-value < 0.05. (**C**) Treatment of hyperglycemic zebrafish with vitamin D or SFN increased the time spent in favorable chamber. Treatment of hyperglycemic zebrafish enhanced the time spent (in seconds) in a favorable chamber in the T-maze. The “***”, “**” & “*” in (**C**) represents a significant difference compared with normal control with *p* < 0.0001, *p* < 0.001 & *p* < 0.05 respectively, and $$$, $$ & $ represents significant difference compared with disease control with *p* < 0.0001 *p* < 0.001 & *p* < 0.05 respectively. “ns” represents “non significance”.

Treatment with vitamin D and sulforaphane assisted hyperglycemic zebrafish to regain learning and memory skills: Hyperglycemia is known to deteriorate learning and memory skills in humans [39]. Hence, first we have assessed the ability of vitamin D and positive control SFN in enhancing learning and memory skills in hyperglycemic zebrafish. Experimentally, “T-maze” based test was conducted according to Kundap et al., and Paudel et al., with minor modifications (for the structure and dimensions of T-maze, refer to Supplementary Figure S1a). Prior studies have shown that normal zebrafish usually spend more time in deep square chamber (favorable chamber) [24,26]. Therefore, we have measured the time spent by zebrafish in the favorable square chamber (Figure 3). Zebrafish with cognition impairment are expected to lose this behavior and spend much less time compared with healthy controls.

Experimentally, first, the total time spent by learning task was determined (Figure 3A). During the initial days of training session zebrafish spent lesser time in the deeper square chamber. For instance, at base line (i.e., day “0”) all zebrafish showed similar time patterns. Normal—56.13 ± 3.46; Disease Control—44.25 ± 9.39; vitamin-D 5 mg/L—45.45 ± 6.66; vitamin-D 20 mg/L—54.95 ± 6.66; and SFN—53.45 ± 7.20. This time has increased significantly after 5-days of training to: Normal—116.53 ± 6.04; Disease Control—113.36 ± 4.49; vitamin-D 5 mg/L—103.30 ± 6.55; vitamin-D 20 mg/L—108.65 ± 8.79; SFN—106.11 ± 8.94.

The time spent by zebrafish in the favorable compartment has decreased from 116.53 ± 6.04 s at day 5 of initial training to 96.33 ± 6.39 s at day 82 in normal controls (Figure 3B). But this decrease was much higher in zebrafish residing in water containing 111 mM glucose, i.e., from 113.36 ± 4.49 at day 5 of training to 66.20 ± 6.85 at day 82. Similar decrease was observed in all the groups demonstrating the reproducibility of the test as well as uniformity across the study animals (Figure 3B). For instance, zebrafish residing in 111 mM glucose containing 20 mg/L vitamin D group showed an initial time of 108.65 ± 8.79 s, which had decreased to 65.52 ± 7.10 (Figure 3B). Similarly, the positive control SFN group also had a residing time of 106.11 ± 8.94 at day 5, which had reduced to 62.13 ± 6.46 s at day 82. Hence, this data concludes significantly that hyperglycemia effects the total residing time in the favorable chamber by decreasing it across the group when compared with that of normal control, representing the long-term effect of hyperglycemia on learning and memory skills.

Treatment of diabetic zebrafish with vitamin D and standard SFN showed a significant improvement in residing time (Figure 3C). For example, at the 7th day of post-treatment, hyperglycemic fish treated with 5 mg/L and 20 mg/L vitamin-D resided respectively, for 76.95 ± 8.065 s and 72.65 ± 6.22 s. Fish treated with SFN had not shown a significant effect on the residing time compared to that of disease control, which had 65.46 ± 5.94 residing time compared with SFN, i.e., 80.96 ± 9.48. At 14th day of post-treatment 5 mg/L and 20 mg/L vitamin-D treated fish showed a residing time of 78.14 ± 6.62, and 83.36 ± 8.07 respectively. At this point, the SFN treated fish showed a significant increase (94.35 ± 10.24) of residing time in favorable chamber when compared with that of disease control 60.98 ± 6.85. At 21st day of post-treatment the residing time in hyperglycemic fish treated with 5 mg/L vitamin-D and 20 mg/L vitamin-D and SFN had shown an increase in the time spent to 89.69 ± 11.14 s, 92.33 ± 9.16 s and 101.7 ± 15.44 s respectively (Figure 3C).

### 3.1. Vitamin D and Sulforaphane Reduced the Latency Time in Hyperglycemic Zebrafish

**Figure 4:**
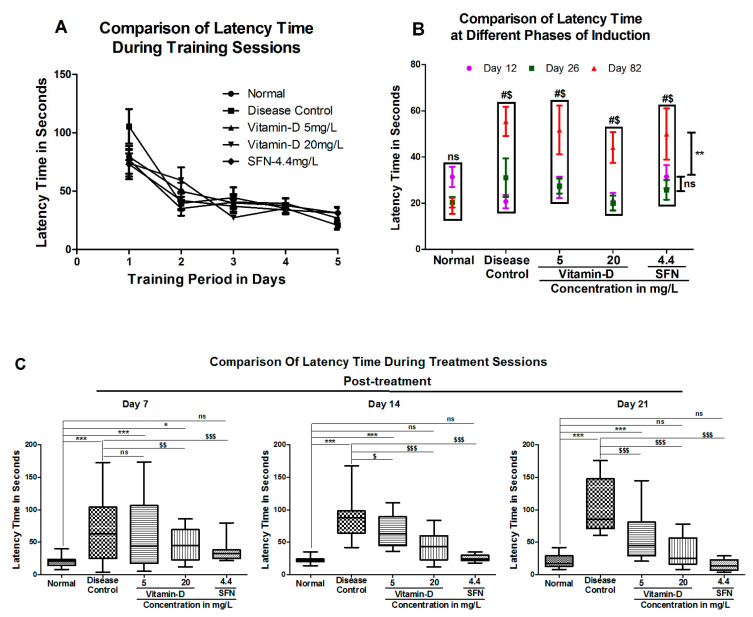
Vitamin D and SFN Could Reduce the Latency Time in Hyperglycemic Zebrafish. (**A**) A 5-days training reduced the latency period in zebrafish: Latency i.e., the time taken to reach the deep square chamber of T-maze, was assessed during the training session in all the groups (*n* = 15) for 5 days. A time dependent decrease was observed with the increase in training time. (**B**) Hyperglycemic environment increased the latency time in zebrafish. Zebrafish growing in 111 mM glucose containing water exhibited ~3 fold higher latency time (at day 82) to reach the deeper square chamber compared with the latency time required at day 12. “**” represents significance between day 12 and day 82 with *p* < 0.001; “#” represents significance in latency time between day 26 and day 82 at *p*-value < 0.05; “$” represents significance in latency time compared with the normal control group at day 82 with *p*-value < 0.05. (**C**) Treatment of hyperglycemic zebrafish with vitamin D and SFN reduced the latency time: Treatment of hyperglycemic zebrafish with 5.0 mg/L and 20 mg/L vitamin D reduced the latency time by 1.5 to 2.0 fold. The data shown represents mean ± SEM with ONE-way ANOVA, followed by Tukey’s post-hoc test using multiple comparisons option. The “***” & “*” represents a significant difference compared with normal control with *p* < 0.0001 & *p* < 0.05 respectively and “$$$”, “$$” & “$” represents significant difference compared with disease control with *p* < 0.0001, *p* < 0.001 and *p* < 0.05 respectively. “ns” represents non-significance.

Latency time is another measure most commonly used to assess the memory function [40]. Providing training to zebrafish for 5 days has reduced the latency time from ~80 s to ~30 s (Figure 4A). With consecutive training sessions, the zebrafish could successfully learn to reach the favorable deeper chamber at much shorter time (Figure 4A). The zebrafish in normal, disease control and treatment groups—vitamin D 5 mg/L and 20 mg/L and SFN (4.4 mg/L) could reach the target respectively in 31.40 ± 4.34 s, 20.73 ± 2.84 s, 26.85 ± 4.61 s, 20.75 ± 3.76 s and 31.44 ± 5.04 s. In conclusion, the 5-days of training to zebrafish reduced the latency time, indicating that the zebrafish had learnt to reach the target chamber much quickly.

Hyperglycemic state has increased the latency time in trained zebrafish from 18.91 ± 3.47 s to 55.40 ± 6.29 s (Figure 4B). For instance, the zebrafish in the vitamin-D 5 mg/L and 20 mg/L could reach the target, respectively, in 51.70 ± 10.53 s and 44.11 ± 6.71 s. The positive control SFN group fish had taken 49.93 ± 11.07 s at day 82. In summary, prolonged hyperglycemic state has increased the latency time in all the fish. Treatment with vitamin D significantly lowered the latency time in a dose dependent manner. Whereas the latency time of disease control group is 102.0 ± 12.70 s, the zebrafish treated with 5 mg/L vitamin D had a latency period of 61.0 ± 11.34 s and the ones residing in 20 mg/L vitamin-D had 33.0 ± 6.69 s at post-treatment of 21st day assessment. The positive control SFN group had a latency period of 14.75 ± 2.47 s (Figure 4C) at 21st day. In summary, vitamin D and SFN mitigated the cognitive impairment caused by hyperglycemia in zebrafish.

### 3.2. Treatment with Vitamin-D Modulated the Expression of Brain Derived Neurotrophic Factor (BDNF) and Acetylcholine Esterase Activity in Zebrafish

**Figure 5:**
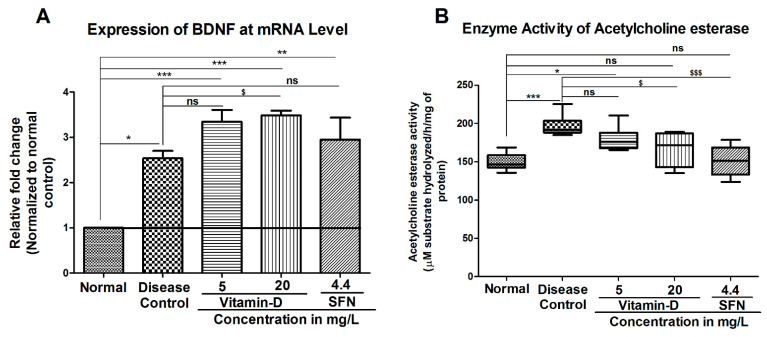
Vitamin D and SFN Modulated the Brain-Derived Neurotrophic Factor (BDNF) and Acetylcholine Esterase Activity. (**A**) Hyperglycemia has induced BDNF expression in zebrafish: A significant increase in the BDNF mRNA was observed in zebrafish residing in 111 mM glucose. Treatment with vitamin D and SFN had no significant difference in BDNF mRNA when compared to disease control. “***”, “**” & “*” Represent the significant differences in BDNF mRNA expression when compared with healthy control group with *p* < 0.0001, <0.001 & <0.05. ‘’$’’ Represents a significant difference in BDNF expression when compared with disease control with *p* < 0.05 and “ns” represents a non-significance compared to disease control. (**B**) Twenty milligram per liter (20 mg/L) vitamin D and SFN reduced the diabetes induced acetylcholine esterase activity. Hyperglycemia significantly increased the activity of acetylcholine esterase in zebrafish. “***” & “*” Represents significant difference in acetylcholine esterase activity between the groups when compared with normal control with *p* < 0.0001 & <0.05 respectively. Treatment with 20 mg/L vitamin D and SFN reduced the hyperglycemia induced acetylcholine esterase activity “$” & “$$$” represents significant difference in acetylcholine esterase activity between the groups when compared with disease control with *p* < 0.05 & <0.0001 respectively. “ns” represents non-significant difference. Data represents mean ± SEM. Data were analyzed statistically by one-way ANOVA, followed by Tukey ‘s post hoc test, considering *p* < 0.05 as significant. The specific activity of acetylcholine esterase was reported as a micromole of thiocholine released per hour per milligram of protein.

In the current study, BDNF expression was increased non-significantly in the 5 mg/L vitamin D group, and significantly in 20 mg/L vitamin-D by 3.34 ± 0.26 fold and 3.48 ±0.10 fold, respectively. The known activator of Nrf2 i.e., SFN showed an increase in the BDNF level non-significantly by 2.94 ± 0.49 fold in the zebrafish brains compared to that of disease control group, which showed a 2.536 ± 0.162 fold increase (Figure 5A).

Acetylcholine esterase is one of the key enzymes, which is known for its ability to induce apoptotic death in pancreatic beta cells [41]. Elevated acetylcholine esterase activity was observed in diabetic subjects compared with healthy volunteers [42]. Therefore, to determine whether AChE is elevated in diabetic zebrafish, the activity of AChE was estimated as detailed in materials and methods and the data in Figure 5B. Analysis of the data demonstrated that AChE activity was significantly increased in the hyperglycemia group (196.5 ± 5.98) when compared with the normal group (149.5 ± 4.61) (Figure 5B). Twenty milligram per liter (20 mg/L) vitamin D but not 5 mg/L vitamin D has significantly decreased the AChE activity to 166.5 ± 8.92 units in diabetic treated zebrafish (Figure 5B). Similarly, the positive control SFN has also reduced the AChE activity to 149.4 ± 5.67 (Figure 5B). In summary, treating hyperglycemic zebrafish with vitamin D and SFN increased the BDNF expression and reduced the AChE activity, suggesting that supplementation of diets with vitamin D can be considered as an important strategy in treating diabetes-induced cognitive disabilities at the early stage of onset.

### 3.3. Vitamin-D and Sulforaphane Have Modulated The Hyperglycemia-Induced Oxidative Stress in Zebrafish

**Figure 6:**
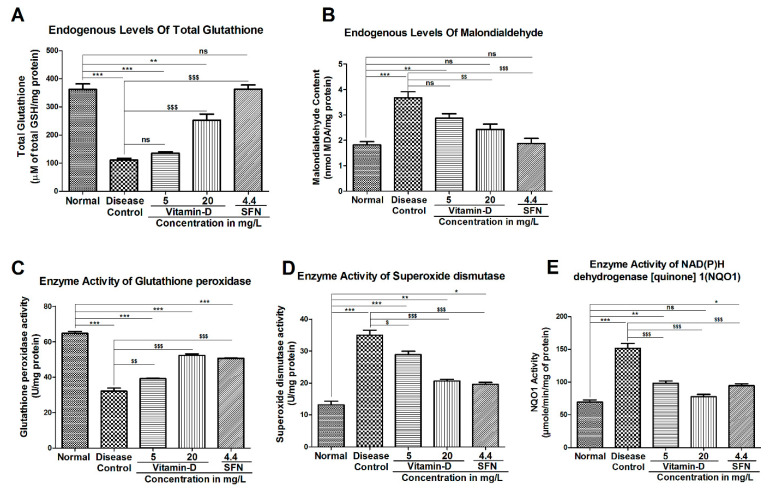
Hyperglycemia-induced oxidative stress markers were modified by vitamin-D and sulforaphane. (**A**,**B**) Vitamin D and SFN could mitigate the hyperglycemia-induced changes in the reduced-glutathione (GSH) and malondialdehyde (MDA) levels in zebrafish. Hyperglycemia has decreased the endogenous glutathione level in zebrafish compared with normal control ones. Treatment with vitamin D and SFN moderately increased the GSH level in hyperglycemia zebrafish (**A**). The oxidative stress marker MDA (a product of lipid peroxidation) has increased in the hyperglycemia zebrafish. Elevated MDA level was decreased by the treatment with vitamin D and SFN (**B**). The “***” & “**” represents a significant difference compared with that of normal control group with *p* < 0.0001 & <0.05. “$$$” & “$$” Represents a significant difference compared with that of the disease control group with *p* < 0.0001 & <0.05. ‘ns’ represents a non-significance. (**C**–**E**) Treatment with vitamin D and SFN modulated the activity of glutathione peroxidase (GPX), superoxide dismutase (SOD) and NADPH quinone oxidoreductase 1 (NQO1) in zebrafish. Hyperglycemic state has reduced the GPX while increasing SOD and NQO1 activity in zebrafish (**C**–**E**). Treatment with vitamin D and SFN has increased the GPX level while decreasing diabetes-induced SOD and NQO1 activity. The data represents mean ± SEM. The results were analyzed by one-way ANOVA, followed by Tukey’s post-hoc test, used for multiple comparisons. The “***”, “**” & ‘’*’’represents a significant difference compared with that of the normal control group with *p* < 0.0001, <0.001 & <0.05. “$$$”, “$$” & ‘’$’’ Represents a significant difference compared with that of the disease control group with *p* < 0.0001, <0.001 & <0.05. ‘ns’ represents non-significance.

At the end of the experiment, the zebrafish were euthanized, and the brain homogenate was prepared to estimate the concentration of lipid peroxidation end products MDA and endogenous antioxidant GSH and various enzymes involved the synthesis and degradation of reactive oxygen species (ROS). Hyperglycemic condition is known to elevate the oxidative stress by increasing glucose auto oxidation, protein glycation and degradation of glycated proteins [43]. Unusually high ROS and a decline in cellular antioxidant defense systems increase insulin resistance and lipid peroxidation, which culminates into cellular damage and the inactivation of cytoprotective enzymes [44]. As a consequence, these oxidative stress events the diabetic complications become more and cause damage to other organ systems [45]. Hyperglycemic state has significantly decreased the glutathione (GSH) from 362.0 ± 19.98 µM in healthy control zebrafish to 110.8 ± 6.25 µM in diabetic ones (Figure 6A). Twenty-one days of treatment with the vitamin-D 5 mg/L marginally increased the GSH level to 135.7 ± 4.58 µM. Increasing the vitamin D concentration to 20 mg/L elevated the GSH level to 253.1 ± 21.12 µM. Treatment with SFN yielded much better effect, as the GSH level has increased to 362.9 ± 14.93 µM (Figure 6A).

Elevated oxidative stress is further confirmed by an increase in the lipid peroxidation product MDA in hyperglycemic zebrafish (Figure 6B). A significant increase in the MDA level from 1.818 ± 0.126 nanomoles/mg total protein in normal control to 3.670 ± 0.24 nanomoles/mg total protein in the diabetic group was observed (Figure 6B). Treatment with vitamin-D at 5 mg/L and 20 mg/L has reduced the hyperglycemia-induced MDA to 2.872 ± 0.169 nanomoles and 2.431 ± 0.207 nanomoles, respectively (Figure 6B). Control SFN could produce a much better reduction in MDA (1.877 ± 0.201 nanomoles/mg total protein).

**Figure 7:**
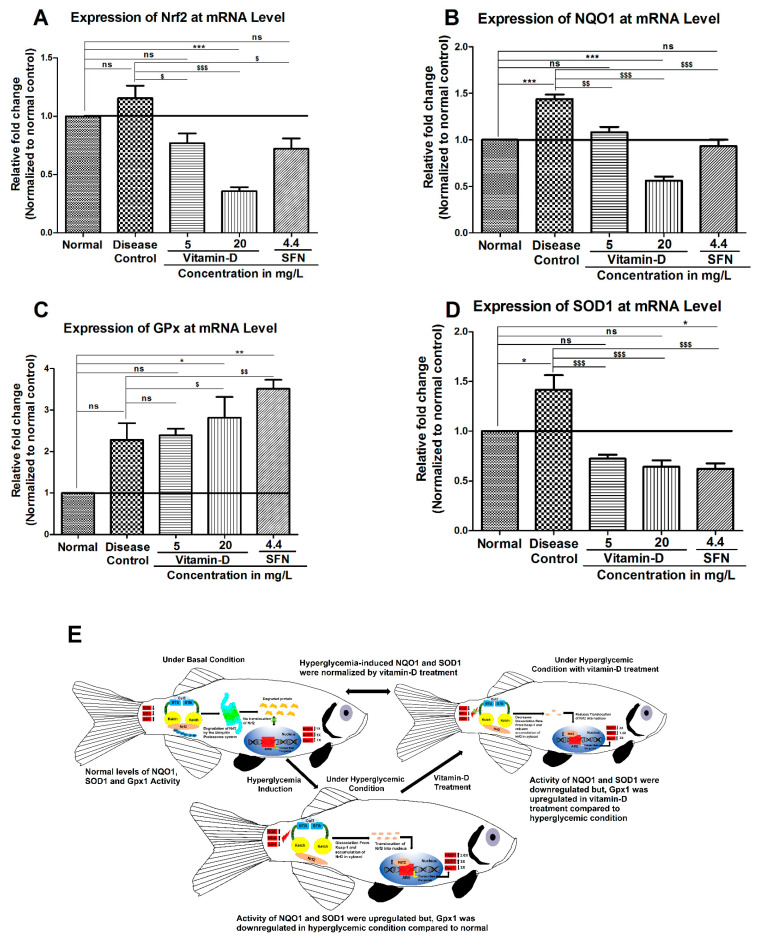
Hyperglycemia and treatment with vitamin D and SFN modulated the mRNA expression of antioxidant genes in zebrafish brains. (**A**) Treatment of hyperglycemic zebrafish has reduced the expression of Nrf2: The hyperglycemia induced Nrf2 expression was reduced by the treatment with vitamin D and SFN. (**B**–**D**) Hyperglycemia has increased the expression of Nrf2 target genes NQO1, SOD and decreased GPx: Correlating with the Nrf2 expression, a significant increase in the Nrf2 target genes NQO1 (**B**), GPX SOD (**D**) and decrease in GPX (**C**) was observed upon the induction of hyperglycemia. Treatment with vitamin D and SFN has reduced the hyperglycemia induced NQO1 and SOD level but not GPX (**B**–**D**). The data represents mean ± SEM. The results were analyzed by one-way ANOVA, followed by Tukey’s post-hoc test, used for multiple comparisons. The “***”, “**” & “*” represents a significant difference compared with normal control with *p* < 0.0001, <0.01 & <0.05 respectively. The “$$$”, “$$” & “$” represents a significant difference compared with the disease control group with *p* < 0.0001, < 0.01 & < 0.05 respectively. (**E**) Effect of vitamin-D on the antioxidant’s enzyme in zebrafish (*Danio rerio*).

Hyperglycemia is known to modulate the antioxidant defense enzymes GPX, SOD and NQO1 [46]. GPx is one of the key enzymes, which utilizes reduced glutathione (GSH) to neutralize peroxides [47]. One of the reasons for elevated oxidative stress in diabetics is the presence of very low GPX. In our study, we have noticed a significant reduction in GPX activity in hyperglycemic zebrafish (Figure 6C). The GPX activity has reduced from 64.79 ± 1.06 units in control zebrafish to 32.07 ± 1.76 units in diabetic fish (Figure 6C). Upon treatment with the vitamin-D 5 mg/L, the GPX activity has increased to 39.18 ± 0.21. Increasing vitamin D concentration to 20 mg/L further induced the GPX activity to 52.26 ± 0.88. The control SFN increased the GPX activity to 50.65 ± 0.19 (Figure 6C). SOD is another antioxidant enzyme involved in mitigating the deleterious effects caused by superoxide radicals [48]. Interestingly, a significant increase in SOD was observed in hyperglycemic zebrafish (Figure 6D). This elevated SOD was reduced by treatment with vitamin D and SFN (Figure 6D). For example, treatment with 5 mg/L and 20 mg/L vitamin D has reduced the SOD activity from 34.96 ± 1.59 in diabetic fish to 28.87 ± 1.06 and 20.60 ± 0.55, respectively (Figure 6D). An increase in NQO1, another antioxidant enzyme, was also observed in hyperglycemic zebrafish (Figure 6E). Treatment of hyperglycemic zebrafish with vitamin D and SFN has reduced the hyperglycemia-induced NQO1 activity (Figure 7E). The NQO1 activity has increased from 69.29 ± 3.38 units in healthy controls to 151.6 ± 7.26 in hyperglycemic zebrafish (Figure 6E). Treatment with vitamin-D 5 mg/L has reduced the elevated NQO1 activity to 98.29 ± 3.45 units while higher vitamin-D (20 mg/L) has further decreased the activity to 77.56 ± 3.49. Control compound SFN could reduce the NQO1 activity to 94.19 ± 3.26 units.

In order to further test and correlate the enzymes (GPX, SOD and NQO1) activity data with that of their expression status, the expression of these enzymes was estimated at mRNA level by qRT-PCR. Analysis of the data showed a significant change in the mRNA of NQO1, GPX and SOD in hyperglycemic zebrafish (Figure 7B–D). Since Nrf2 controls the expression of these enzymes, we have measured the Nrf2 expression in hyperglycemic zebrafish and compared with the healthy control group. As predicted, a visible increase in the Nrf2 expression was observed in hyperglycemic fish (Figure 7A). Interestingly, but unpredicted, the known activators of Nrf2 signaling viz., SFN and vitamin D have reduced the expression of Nrf2 and its target genes NQO1 and SOD (Figure 7A,B,D). In contrast to this, the level of GPX has increased by the vitamin D and SFN in hyperglycemic zebrafish (Figure 7C). In the current study we shown that under hyperglycemia condition antioxidants like NQO1, SOD1 were upregulated and Gpx1 was down regulated and upon vitamin-D treatment this conditioned were normalized (Figure 7E). Further studies measuring the expression of these enzymes at protein level are warranted to correlate the activity of these enzymes with expression. 

Vitamin D reduced oxidative stress by promoting the expression of Nrf2 in neuronal cell line SK-N-SH: Since vitamin D showed protective effect from hyperglycemia-induced oxidative stress, we have hypothesized that it might be triggering the antioxidant mechanisms in the cells through either (a) promoting the expression of antioxidant proteins or (b) by inactivating the inhibitory molecules that negatively acts on antioxidant defense system. To find this, we have first tested whether vitamin D treated neuroblastoma cell line SK-N-SH express high levels of Nrf2 compared with untreated cells (Please refer Supporting Methods section). Treatment with 31.25 µM vitamin D has increased Nrf2 expression by ~2.0 fold compared with untreated cells (Supplemental Figure S2a). The known activator of Nrf2 i.e., sulforaphane, increased the expression by ~3.2 fold (Supplemental Figure S2b). Even though it is now confirmed that vitamin D is enhancing the expression of antioxidant Nrf2 in the in vitro studies, it is still unknown how vitamin D is increasing the expression of Nrf2 in cells.

**Figure 8:**
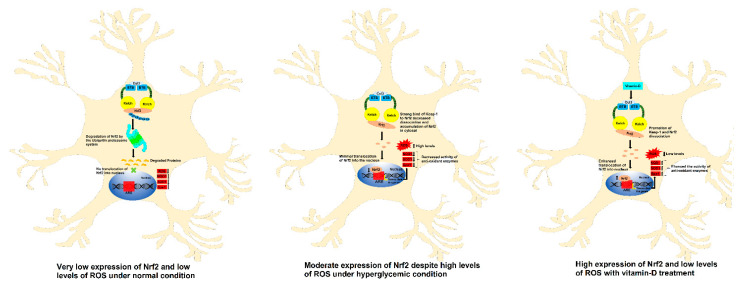
Effect of vitamin-D on the Nrf2 and antioxidants enzyme in SKNSH cell line.

Further, to determine whether vitamin D-induced Nrf2 is functionally active, the cellular oxidative stress was determined by measuring ROS levels as detailed in the Supporting Methods section. Vitamin D treatment reduced the cellular oxidative stress suggesting that the vitamin D not only enhanced the expression of Nrf2 but also increased its function (Supplemental Figure S3). Moreover, a significant increase in the activity of a direct target of Nrf2, i.e., NQO1 was also observed upon treatment with vitamin D (data not shown). There by considerably concluding that in the human cell line SKNSH vitamin-D treatment upregulated the antioxidants level thereby reducing the oxidative stress (Figure 8).

Under normoxic conditions, Keap1 inactivates Nrf2 by binding [49]. Therefore, we have tested whether vitamin D inhibits the binding of Keap-1 to Nrf2 using in silico docking approach. Experimentally, vitamin D3 was evaluated for its binding affinity towards Keap-1 (4IQK) and the results represented in Table 2. Vitamin D exhibited the highest binding affinity with a docking score of −56.45 towards 4IQK. The known activators of Nrf2 viz., Sulforaphane, Pterostilbene and Bardoxolone showed a docking score of −24.82, −37.79 and −16.79 towards Keap-1(4IQK) respectively. Based on these observations, we hypothesize that vitamin D-mediated activation of Nrf2 is likely due to its strong binding strength to the Keap-1 [50].

**Figure 9:**
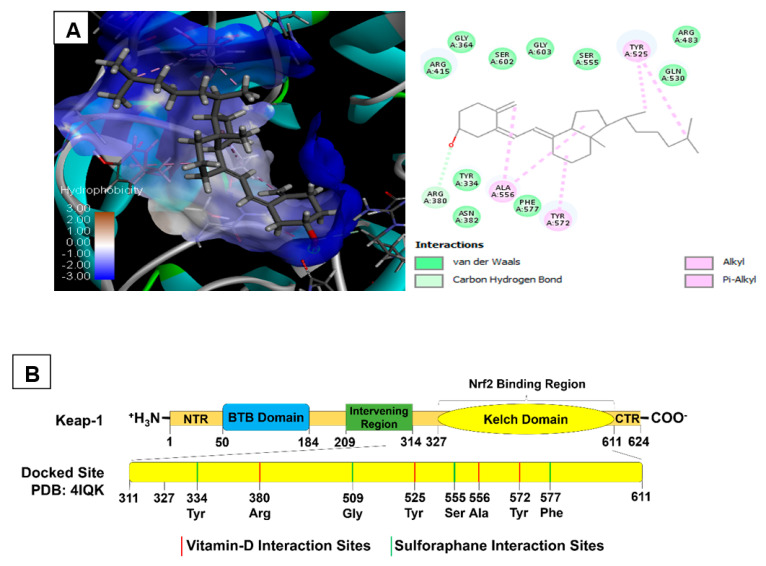
(**A**,**B**) Molecular docking of cholecalciferol to Keap1 (4IQK). Cholecalciferol showed a better binding affinity towards Arg380 in the carbon hydrogen bond. The target receptor is attached to cholecalciferol through Ala556, Tyr525, and Tyr572.

Analysis of the best docking poses showed the most interacting residues in the active sites of Keap-1 and cholecalciferol are shown in Figure 9 and Table 2. Further analysis of the interactions between cholecalciferol and Keap-1 revealed that the H atom (polar H interaction) of ligands were bound via carbon hydrogen bond to Arg380 with the contact distance of 2.73 Å whereas the aromatic and benzene rings were attached via alkyl (hydrophobic interaction) to Ala556, Tyr525 and Tyr572 with the contact distance of 3.57 & 5.30, 4.17 & 5.19 and 4.47 Å, respectively. Cholecalciferol and its interactions with Keap-1 in 3D and 2D poses are shown in Figure 9A,B. Interactions of other molecules with Keap-1 are represented in Supplemental Figure S4.

## 4. Discussion

Diabetes is a complex disorder, which modifies protein structure and function and is responsible for various diabetic complications. For example, glycation of proteins, peroxidation, and oxidation reactions are known to cause microvessel damage [51]. Uncontrolled diabetes can also damage the mitochondria and induce defects in oxidative phosphorylation reactions and promote the formation of advanced glycation end products (AGES) enhances polyol pathway and hypoxic conditions. In addition, poor glycemic control causes alterations in lipoprotein metabolism, and induces protein kinase C activity and the release of growth factors and cytokines. Elevated oxidative stress is another key feature of diabetes-induced metabolic alteration [52]. Although oxidative stress appears as one of the key metabolic events associated with severe diabetes, the precise mechanisms by which oxidative stress several complications, in particular cognition impairment, are still not well understood [45]. Hence, in the current study, we have first determined the impact of hyperglycemia-induced cognitive dysfunction in zebrafish and tested whether treatment with vitamin D could mitigate the complications induced due to hyperglycemia. Zebrafish have been used by many investigators to study the cognitive functions as it has many similarities in the neurochemical properties and basic layout of the brain [53].

Zebrafish have high nucleotide sequence homology and functional similarities with mammalian genes [54]. For example, Kudap et al., 2017 showed epilepsy-induced cognition impairment in adult zebrafish [24]. Jonas et al., 2016 tested zebrafish of different ages for associative learning spatial memory and analyzed the levels of oxidized lipids and proteins as well as lipofuscin. This study suggested that young zebrafish at the age of 1 year could successfully complete learning tasks, but cognitive abilities were significantly impaired in older animals. This study concluded that enhanced oxidative stress might be contributing to cognitive impairment in the aging zebrafish. Results of our study are in line with the oxidative stress level, as the diabetic fishes exhibited higher oxidative stress compared with normal [55]. Bagle S et al., 2021 induced memory deficit in the adult zebrafish by giving Scopolamine. This study showed that apocynin could protect zebrafish from scopolamine-induced memory deficit. Similar to this, in the current study, sulforaphane and vitamin D protected zebrafish from diabetes-induced memory impairment [56]. Results of our study showed that hyperglycemic zebrafish are not capable of attaining good performance in associative- and spatial-learning tasks compared with the zebrafish in the normal control group. Similar to our study, Rajan et al., 2020 reported good associative learning and memory establishment in healthy zebrafish but not in zebrafish residing in a hyperglycemic environment [57].

Hyperglycemic zebrafish were found to be less motivated, depressed, and did not show interest in exploring the maze compared with the control group in finding the target arm, suggesting that training the fish in the hyperglycemic group was not successful compared with that of fish in the control group. In summary, hyperglycemia affected the learning memory of the zebrafish. Similar observations were also reported by Santos et al. 2018, who had observed anxiety like behavior, stress and depression in the zebrafish residing in hyperglycemic conditions compared with the one residing in non-hyperglycemic water [58]. Allen et al. reviewed 10 studies exploring the relationship between DM and cognitive functions (9 population-based studies and 1 case control study) and found evidence of a possible link between DM and both cognitive decline and dementia [59]. Later, a systematic review by Biessels et al. Provided strong evidence of an increased risk of dementia in patients with DM [60].

Many epidemiological studies have shown that the risk of DM and cognitive dysfunction increases with vitamin D deficiency [61,62]. A study by Van der Schaft et al. reported worse cognitive function and increased risk of dementia with low vitamin D in 18 (72%) of the 25 cross-sectional studies [63]. Few other studies have reported increased cognition decline in patients with vitamin D deficiency [64,65,66]. But these findings need support from well-designed randomized control trails. In the current study, we showed a significant decrease in the decline in cognition impairment upon the administration of vitamin D.

The Brain-Derived Neurotrophic Factor (BDNF), which is one of the major neurotrophins produced in the brain, helps in the survival of neurons, synaptic plasticity, and memory. Several studies have shown that BDNF is decreased in diabetic subjects compared with healthy non-diabetic volunteers [67]. Moreover, studies have also shown that enhancement of BDNF signaling cascade alleviates cognitive impairment in diabetic rats [68] and protects against neuronal apoptosis and synaptic plasticity dysfunction under hyperglycemic condition [69].

Hyperglycemia is known to increase the level of ROS, which leads to the inflammation or neuronal damage in the brain. Elevated ROS is also known to decrease the BDNF level in diabetic subjects [70]. Even we have observed that diabetes has decreased the BDNF mRNA expression in zebrafish. Since vitamin D deficiency has been associated with insulin resistance, pathogenesis of T2DM and cognitive impairment, we have assessed the level of BDNF in diabetic zebrafish treated or not treated with vitamin D. Data from our study has shown that vitamin D treatment has increased the BDNF level in diabetic zebrafish.

Acetylcholine esterase (AChE) plays a crucial role in learning and memory process [71]. Elevated acetylcholine (Ach) in the synaptic cleft plays a key role in the learning- and memory functions [72]. Supporting these observations, changes in AChE structure and activity have been reported in diabetic individuals [73]. Similar to these observations, even we have found an increase in the AChE activity in animals treated with 111 mM glucose. Further, AChE activity was decreased in vitamin D and SFN treated groups. Similar to our observations, a previous study reported that SFN treatment decreased the age-related neuro-inflammation. Authors of this study showed that SFN significantly improved the hyperglycemia-induced alteration in the T maze test as well as in the AchE activity inhibition test indicating that sulforaphane could mitigate the complications observed in neurological disorders. Since SFN is a known activator of Nrf2 and Nrf2 is known to reduce oxidative stress and inhibit neuroinflammation and neurodegeneration, we have hypothesized that vitamin D treatment might also protect cells by reducing cellular oxidative stress and inflammation [74]. We have demonstrated that vitamin D treatment significantly reduced the AchE level in hyperglycemic zebrafish. Similar to our data, a study reported reduced hippocampal AchE level in high-fat diet induced memory impairment model in rats upon feeding with vitamin D [75].

Lipid peroxidation, which is represented by elevated MDA level, is another characteristic reported widely in diabetic subjects [76]. Increase in the plasma MDA has been reported in diabetic zebrafish by many investigators [77]. Hyperglycemia promotes ROS mediated lipid peroxidation via nonenzymatic and auto-glycation pathways [78]. Hyperglycemia reduced the GPx activity as an adaptive response, as demonstrated by our study as well as by many other investigators [79]. SOD is another key enzyme involved in controlling oxidative stress. Low SOD activity in diabetic animals, as observed in this study, could be due to glycation of SOD by elevated glucose. A prior report showed that nearly 50% of SOD in diabetics is in its inactivated state due to glycation. Furthermore, excess hydrogen peroxide, which is produced due to lipid peroxidation and autooxidation of glucose, also contributes to higher the SOD activity in T2D [80]. In our study SOD activity has decreased in vitamin D treated zebrafish, suggesting an endogenous adaptive response.

Vitamin D treatment reduced the hyperglycemia-induced decline in the Nrf2 level in the zebrafish. Elevated vitamin D3 up-regulated multiple genes and functional pathways involved in synaptic vesicle trafficking and neurotransmission [81]. Several genes, including synaptojanin 1 and synaptotagmin 2, contain a potential vitamin D response element in their promoter region and may be subject to direct modulation by vitamin D. A significant improvement in the leaning memory was observed with vitamin D in a dose depend manner compared with hyperglycemic control. Furthermore, vitamin D supplementation has shown the effect on antioxidant enzymes and molecular pathways, which further helps to scavenge the ROS.

## 5. Conclusions

The results of our study showed a decline in cognition in zebrafish residing in hyperglycemic condition compared with the ones residing in normal conditions. We have shown that decline in cognition is in part due to the selective inhibition of GPx activity (as observed in zebrafish), or the mitigation of even other antioxidant enzymes SOD or NQO1 (as observed in human neuronal cell line), leading to elevated oxidative stress. Activation of Nrf2 by vitamin D, through its ability to strongly bind to Keap1, promoted the activity of target enzyme GPx in zebrafish or all other antioxidant enzymes that include SOD and NQO1 as observed in neuronal cell line. Vitamin-D supplementation not only reduced the hyperglycemia-induced oxidative stress but also mitigated the cognition impairment. In summary, we propose vitamin D as a potential molecule to consider for the treatment of diabetes-induced cognition impairment. Testing vitamin D in higher animals is immediately required to further qualify the observations in this study.

## Figures and Tables

**Table 1:** Primer sequences used, and the expected product size of target genes in the RT-PCR reaction.

Gene	Forward Sequence (5′ → 3′)	Reverse Sequence (5′ → 3′)	Product Length (bp)	Annealing Temperature (°C)	Accession Number
NRF2	GAGCGGGAGAAATCACACAGAATG	CAGGAGCTGCATGCACTCATCG	82	65	AF057040
GPX1	CAGATGAACGAGCTCCACAG	CCATTCACTTCCAGCTTCTCC	185	58.5	NM_001007281
NQO1	CGAGATGTTGCAGTTCAGGC	ATCGACCCTCTTTCCATGCA	175	53	NM_205542
SOD1	CGCACTTCAACCCTCATGAC	TGAATCACCATGGTCCTCCC	137	50	NM_131294
BDNF	CGCTCACCATGTCATCCAAC	TCTGCGATATTCGTCCGCTC	173	58.5	NM_131595.2
β-ACTIN	CGAGCAGGAGATGGGAACC	CAACGGAAACGCTCATTGC	102	60	AF057040

**Table 2:** Docking score, nature of interaction(s), and the key amino acids involved in binding of selected Nrf2 activators.

Selected Biomolecules	Protein & PDB ID	Docking Score	Nature of Interaction(s)	Active Site Amino Acids
Cholecalciferol	Keap1 (4IQK)	−56.45	Carbon Hydrogen Bond	Arg380
			Alkyl	Ala556, Tyr525, Tyr572
Sulforaphane		−24.82	Carbon Hydrogen Bond	Ser555, Gly509
			Pi-sulfur	Phe577, Tyr334
Pterostilbene		−37.79	Carbon Hydrogen Bond	Arg530, Gln530
			Pi-Alkyl	Tyr572, Tyr525
			Conventional Hydrogen bond	Ser336, Ser508
Bardoxolone		−16.79	Conventional Hydrogen bond	Tyr572, Asn382, Arg380, Arg415
			Pi-Alkyl	Tyr334, Arg415

## Data Availability

Data is contained within the article and Supplementary Materials.

## Supplementary Materials

The following are available online at https://www.mdpi.com/article/10.3390/antiox11112114/s1, Figure S1: Pictorial representation of T-Maze used for the behavioral assessment, Figure S2: Treatment with Vitamin-D enhanced the expression of Nrf2 in SK-N-SH cell line. Figure S3: Treatment with Vitamin-D effectively reduced the endogenous ROS in SK-N-SH cell line. Figure S4: Molecular docking of Sulforaphane (A), Pterostilbene (B) and Bardoxolone (C) to Keap1 (4IQK).
