# Supplementary material for: Vitamin D Mitigates Hyperglycemia-Induced Cognition Decline in Danio rerio (Zebrafish) through the Activation of Antioxidant Mechanisms
Source: Antioxidants (Basel). 2022 Oct 27;11(11):2114. doi: 10.3390/antiox11112114 (PMC9686813; doi:10.3390/antiox11112114)
Supplement: Supplementary file 1 [file antioxidants-11-02114-s001.zip › antioxidants-1977587-supplementary.pdf]

Assessment of Learning and Memory:

Assessment of learning skills and memory test by T-maze: Learning and memory assessment tests were performed by using a T-maze as referenced [24,26]. Briefly, T-maze consists of a long arm with the length of 457.2mm, and two short arms of length 304.8mm each. The width of the arms is 101.6mm. One of the short arm was provided with a deep square cuboid compartment of 228.6mm, which serves as favourable area for fishes to spend more time as it has the deeper and wider space compared to the other arms of the maze (**Supplemental Figure S1a**). The behaviour was assessed in a room, which was provided with camera for recording the movements of the fishes during the assessment.

Training sessions: The training sessions were carried out with one trial each day for five consecutive days. The first day of training was started by placing the fish in T-maze's long arm and allowed to explore the tank for 180 seconds; subsequently the fish were shifted to the home tank after the trial. On day two, fishes were trained to reach the deeper square chamber. Following consecutive days fishes were allowed in the tank to assess their ability to reach and spend time in the deeper square compartment during the 180 seconds testing time. The time taken to reach the deeper square compartment was considered as latency time. In addition, total time spent in the deeper square compartment was recorded manually.

Induction of hyperglycemia and determination of blood glucose levels: Hyperglycemia was induced by a method previously described by Capiotti KM et al [11]. Procedurally, 20 adult zebrafish were placed in a 3L tank containing 111mM glucose solution for 14 days at room temperature, and feeding was continued twice a day. Glucose solution was exchanged with freshly prepared ones every day to avoid contamination with any opportunistic microbial growth.

To determine the blood glucose levels in the animals, first the zebrafish (6 each) were starved for 12hours and placed in a tank with no glucose for 10-15mins to remove the adherent glucose which otherwise might cause over estimation. The animals were euthanized by hypothermia induction to avoid the variability during the determination of glucose. Immediately after euthanasia the tail was cut and blood glucose reading was determined by placing the glucose strip (Dr. Morepens) directly to the docked tail end. Upon confirmation of hypergylcemia, based on blood glucose level, the remaining animals in each group were continued in the 111mM glucose solution till the completion of the study.

Treatment with Vitamin-D and Sulforaphane

Acute toxicity assessment of vitamin-D: The concentration of vitamin-D, which causes toxicity was determined as per the OECD guidelines. Experimently, first the zebrafish (6 fishes from each group) were acclimatized for 7 days; followed by placing the fish in a solution containing vitamin-D ranging from 6.25mg/L to 100mg/L for 96h. During the exposure period, the animals were observed for any impairment in the swimming pattern or mobility loss and mortality at 24h, 48h, 72h and 96h. Mortality (100%) was observed at 100mg/L vitamin D at 24hours; but, at 50mg/L about 50% mortality was observed with impaired swimming patterns and immobility (at 48hours). No signs of impairment in swimming pattern or mortality were observed at 6.25, 12.5 and 25mg/L even at 96hours of period. Hence, 20mg/L vitamin D was considered as the highest treatable dose. During the entire treatment period, the animals were kept in glucose solution to maintain the hyperglycemic state. The fish were kept in vitamin-D containing solution in the dark cycle of the study.

Gene expression analysis by quantitative real time RT-PCR (RT-qPCR): Expression of genes at mRNA level was quantitated by performing RT-PCR as detailed earlier [32]. First, total RNA was isolated using TRIZOL reagent (Invitrogen) according to the manufacturer's instructions. The isolated RNA was quantified using Nanodrop (Denovix) and 1.0µg of RNA was used to perform cDNA synthesis using VERSO reverse transcription system (Invitrogen). Quantitative PCR was performed using SYBR green II master mix (TAKARA) to determine the expression of target genes (**Table 1**). The reaction mixture (20µL) consists of 10µL of SYBR green master mix, 5µL of cDNA and 5µL of specific primer mix (forward and reverse). The amplification program steps include initial denaturation at 95^{0}C for 7mins followed by denaturation at 95°C for 20secs, annealing at respective temperatures for 30secs and extension at 72^{0}C for 25secs.

Glutathione Peroxidase Activity (GPx) Assay: GPx acitivity assay was performed by incubating 140µL of 1X assay buffer with 20µL of 10X reaction mixture containing Glutathione Reductase, GSH+NADPH mix and 50µg total protein (in a total volume of 20µL). For standard, 20µL of standard Glutathione Peroxidase enzyme was added instead of sample. Twenty microliters of 1X assay buffer was added to the control and sample wells. The reaction was initiated quickly by adding 20µL of Cumene Hydroperoxide to each well. The absorbance at 340nm was measured at every 45 seconds over a period of 30 minutes. The rate of decrease of absorbance at 340nm in the background is subtracted from that of the samples or standard to obtain the net rate of decrease of absorbance. Next, the Glutathione Peroxidase activity was calculated by comparing the data of samples with that of standard enzyme.

Superoxide Dismutase Activity (SOD) Assay: Activity of SOD was determined by loading 25µL of serially diluted standards and samples (50µg protein) in to a 96 well plate. One hundred and fifty microliters of (150µL) of master mix containing 10X SOD buffer, WST-1 reagent, Xanthine oxidase and distilled water was added to all the wells. To initiate the reaction, 25µL of 1X Xanthine solution was added and the optical density was read at 450nm every minute for 10 minutes at room temperature using multimode plate reader. The collected data were used to calculate the percentage inhibition of formation of WST-1 formazan.

NAD(P)H Quinone Oxidoreductase 1 (NQO1) Activity Assay: Briefly, NQO1 activity was determined by using 100µg total protein. Two separate NQO1 cocktails were prepared by mixing Tris, BSA, Tween-20, FAD, Glucose-6-phosphate, Glucose-6-phosphate dehydrogenase, NADP, Menadione, MTT with and without Dicoumarol. Forty microliters sample was loaded into 96 well plate and 200µL of NQO1 cocktail mixture was separately added. The absorbance was measured at 562nm for every 60 seconds for 30 minutes. For additional information about the concentrations of each reagent please refer Prochaska HJ *et al*. [35]

Estimation of Total Glutathione (GSH): The amount of glutathione in the samples was measured by loading 20µL of total protein (50ug) into a 96-well plate followed by incubating with 60µL of NADPH solution (0.66mg/mL) for 30-60seconds at room temperature with gentle agitation. To the incubated samples, 120µL of DTNB (60µL of 0.66mg/mL) and glutathione reductase (60µL of 3.3U/mL) mixture (ratio-1:1) were added and the absorbance was immediately read at 412nm in a kinetic mode for every 60s upto 4mins. The concentration of total GSH in the samples was calculated against the GSH standards and represented as micromolar GSH per mg total protein.

Estimation of Acetylcholine Esterase in Zebrafish brain: Procedurally, first, the rate of hydrolysis of acetylthiocholine (ACSCh, 0.8 mM) in 2.0ml assay solution containing 100 mM

phosphate buffer, pH 7.5, and 1.0 mM DTNB was estimated. Samples containing protein and the reaction medium were preincubated for 10 min at 25ºC followed by initiating the reaction by the addition of substrate. The hydrolysis of substrate was monitored by the formation of thiolatedianion of DTNB at 412 nm every 30s for 2 and half min (150 seconds). The linearity of absorbance was determined; and AChE activity was calculated in micromoles of thiocholine (SCh) released per minute per milligram of protein.

Lipid Peroxidation Assay: Lipid peroxidase activity was determined by first incubating the 80 µL of brain homogenate was mixed with 160µL of 10% TCA and centrifuged for 10 min at 10,000 Xg. Then, 100 µL of separated mixture was added to 100 µL of 0.67% thiobarbituric acid (TBA) and heated at 100ºC for 30 min. The TBARS level was measured spectrophotometrically at 532 nm using a microplate reader. Malondialdehyde (MDA) was used as a standard. Results were expressed as nmol MDA/mg of protein.

Note: All enzyme assays were performed in triplicates. Total protein content was measured by the bicinchoninic acid (BCA) method using Pierce™ BCA Protein Assay Kit. Bovine serum albumin was used as a standard.

Methods related to cell culture and treatment:
Human neuroblastoma cell line SK-N-SH was procured from National Centre for Cell Sciences (NCCS, Pune, Maharashtra, India). The cells were cultured in Eagle's Minimum Essential Medium (MEM; HiMedia Laboratories, Mumbai, Maharashtra, India) supplemented with 10% (vol/vol) fetal bovine serum (FBS; Gibco Life Technologies), 1% penicillin-streptomycin (Gibco Life Technologies, Grand Island, NY, USA) and 1% L-glutamate (Gibco Life Technologies). The cell line was grown in a carbondioxide incubator maintained at 5% CO_2 and 95% relative humidity (95% RH) at 37°C.

Immunofluorescence: SK-N-SH cells were cultured in 8-well chambered slide (IBIDI, Gräfelfing, Germany) at $1X10^5$ cells per well (300µL) and incubated in a carbon dioxide incubator maintained at 5% CO_2 at 37^0C. Cells were treated with vitamin-D (31.25µM) and sulforaphane (5µM) for 48hours. Post-treatment, the cells were fixed with 4% paraformaldehyde in PBS (100µL) for 10mins, followed by washing with PBS. The fixed cells were permeabilized using 0.2% Triton-X100 prepared in PBS (100µL) for 10mins. The cells were washed 3 times with 1X PBS containing 0.1% Tween-20 (200µL) and the non-specific sites were blocked by using 10% goat serum in 1X PBS-T (100µL) for 1hour. After blocking, the fixed cells were incubated with rabbit anti-human primary antibody designed to detect Nrf2 (Proteintech Group, Rosemont, IL, USA). The antibody was diluted (1:400) in blocking buffer and incubated (100µL) at 4^0C overnight. Primary antibody was aspirated and washed thrice with 1X PBS-T (200µL) followed by incubation with anti-rabbit secondary antibody conjugated with FITC (1:250, Sigma, St. Louis, Missouri, USA) (100µL). The incubation was carried out at room temperature for 1hour in dark. The cells were washed thrice with 1XPBS-T (200µL) and subsequently incubated with 1µg/mL DAPI (100µL) (Sigma, St. Louis, Missouri, USA) at room temperature for 2mins in dark. The leftover solutions were aspirated and washed twice with 1XPBS-T, and mounted using anti-fading mounting medium (20µL) (IBIDI). Finally, the cells were visualized using fluorescence microscope (Olympus, Shinjuku, Tokyo, Japan) and images (were captured at four different microscopic fields per well) were quantified using imageJ software and represented as fluorescence intensity.

Quantitative Assessment of Reactive Oxygen Species by H_2DCFDA Method: About 70% confluent culture was trypsinised and plated in a 96 well plate at $1X10^4$ cells per well in 100µL complete medium and incubated in a carbondioxide incubator maintained at 37^0C with 5% CO_2. Later, cells were treated with vitamin-D (31.25µM) and Sulforaphane (5µM) for 48hours. Upon completion of the treatment, 10µM of H_2DCFDA (Sigma, St. Louis, Missouri, USA) was added to the wells and incubated at 37^0C for 30 minutes. The media was

aspirated and washed twice with DPBS (100µL) and fluorescence was measured at Excitation 485nm/ Emission 525nm in a multimode plate reader (Perkinelmer, Waltham, Massachusetts, USA). The data was represented as relative fluorescence units (RLU).

Molecular docking of vitamin D3 and known activators of Nrf2:

Ligand preparation: In this study, Cholecalciferol (Vitamin D3) and known activators of Nrf2 viz., Sulforaphane (SFN), Pterostilbene (PTS) and Bardoxolone (CDDO) were docked to assess and compare the binding strengths each of these molecules to Keap-1 protein (Figure-8A & B). PubChem was used to obtain the structure-data-file (SDF) of the compounds and *Open Babel* software (Version:2.4.1) was used to convert individual SDF file to combined SDF file. The combined SDF file was further subjected to ligand preparation protocol in discovery studio to obtain possible tautomeric- and ionized structures while fixing the valency.

Receptor preparation: The Protein Data Bank (PDB) file of the receptor was downloaded from Research Collaboratory for Structural Bioinformatics (RCSB) PDB (www.rcsb.org). The 3D structures of the Keap-1 target proteins were availed from protein data bank with the PDB ids: 4IQK.

Before receptor preparation, the Keap-1 protein was processed by removing the (a) crystal cell, (b) unwanted chains, (c) ligands or molecules in the binding site and (d) water molecule. After which protein preparation was carried out using protein preparation protocol in DS, which includes addition of hydrogens and building loops.

Docking process: Docking was done by using C-docker algorithm in Discovery studio, which works on the principle of simulated annealing. Inbuilt scoring function to generate -C docker energy and -C docker interaction energy. These values vary based on the interactions of ligand molecule (known activators of Nrf2) and the amino acid residues involved in the binding site of Keap1. These interactions can be favourable and/or unfavourable. Subsequently, we calculated the binding energy of the ligand-protein complex using the “calculate binding energy” protocol in discovery studio. Lower the binding energy better the complex stability. The interaction between the ligands and receptors was identified utilizing the 2D and 3D interactions of the docking by Discovery studio visualizer.

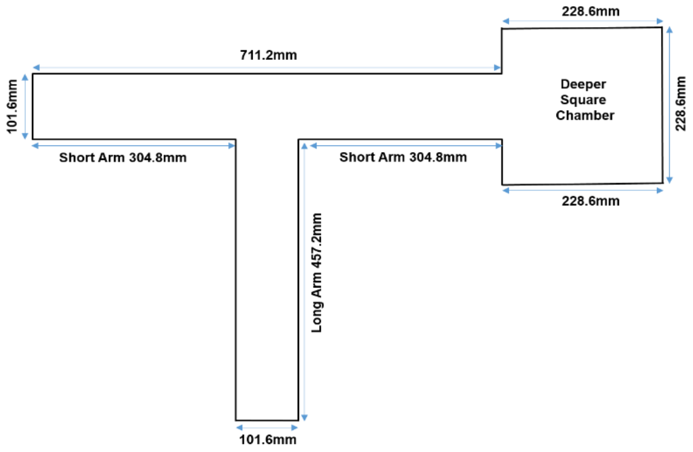

Supplemental Figure S1: Pictorial representation of T-Maze used for the behavioural assessment

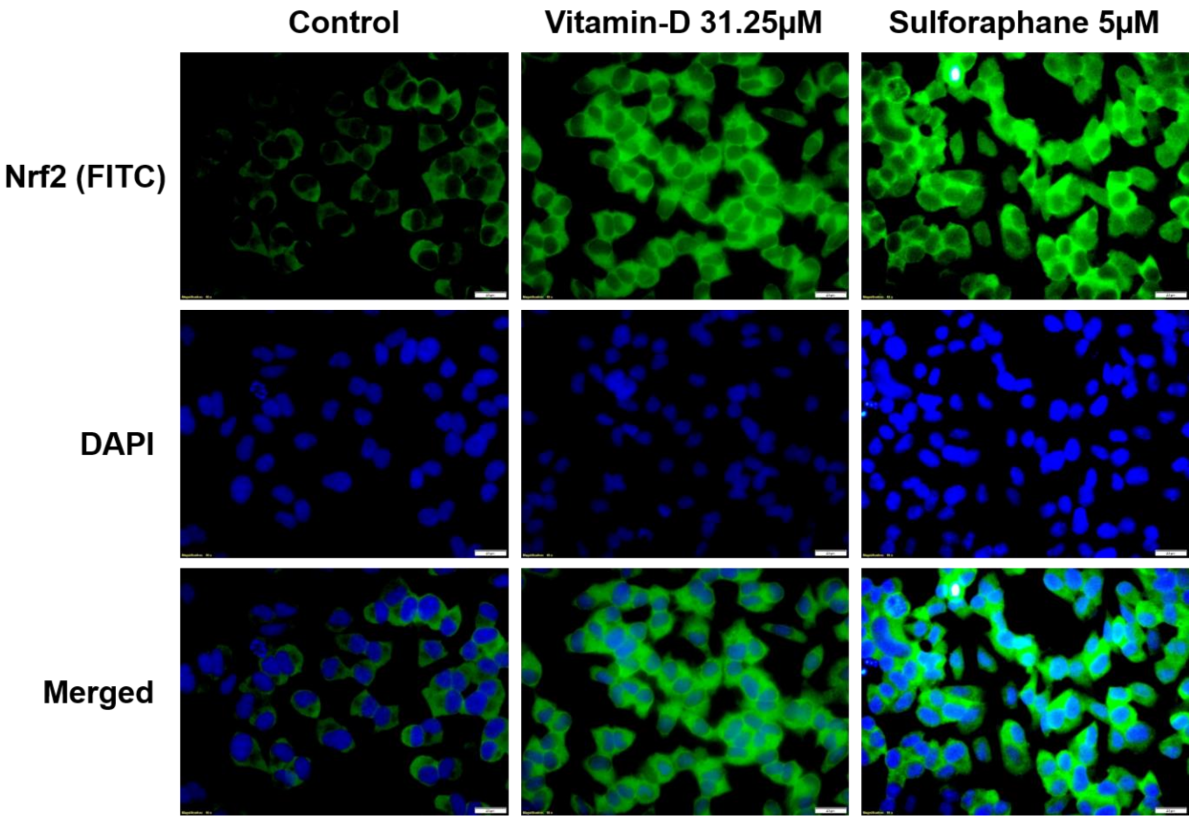

(a)

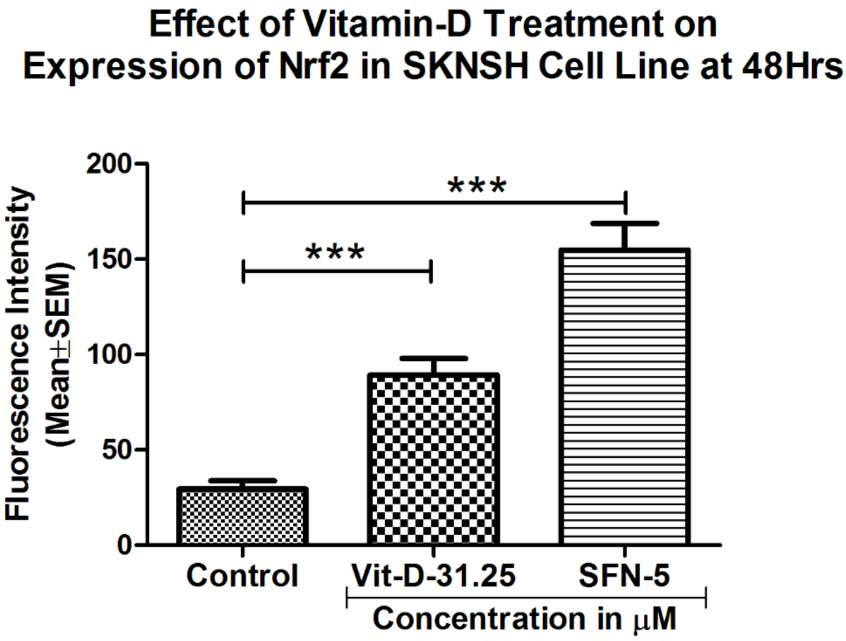

(b)

Supplementary Figures S2a & S2b: Treatment with Vitamin-D enhanced the expression of Nrf2 in SK-N-SH cell line. Analysis of expression of Nrf2 in the SK-N-SH by immunofluorescence showed a significant increase with 31.25μM vitamin-D treatment compared to that of untreated control. Intensity measurements revealed that there was a

significantly higher expression of Nrf2 in the vitamin-D treated cells with significant p-value of P<0.0001 compared to that of control.

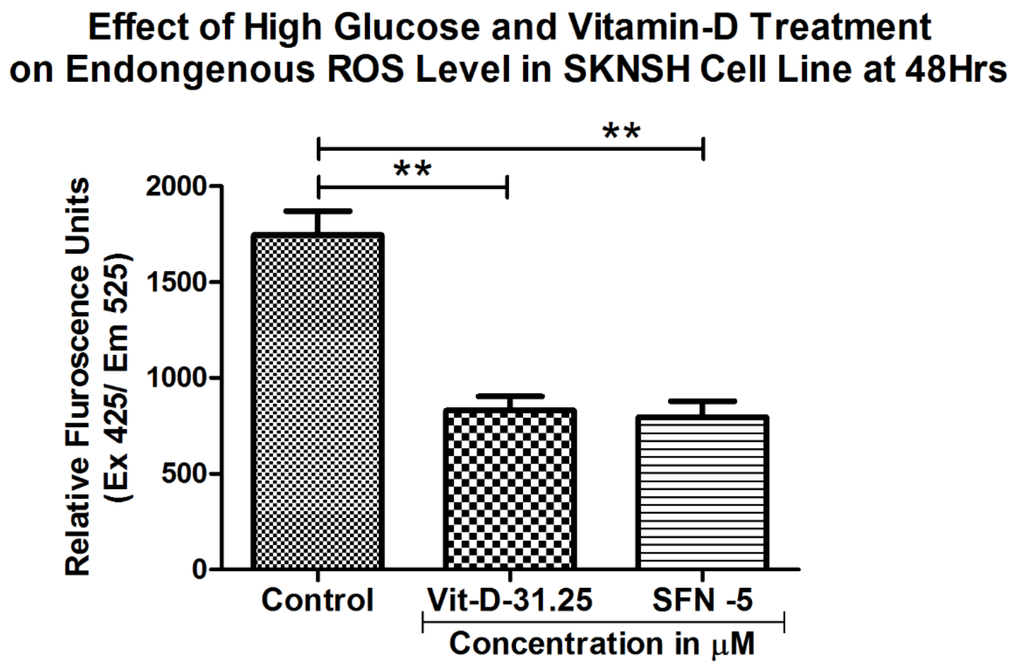

Supplementary Figure S3: Treatment with Vitamin-D effectively reduced the endogenous ROS in SK-N-SH cell line. Detection of ROS using H_2DCFDA method showed a significant decrease in the ROS upon treatment with vitamin-D at 31.25µM compared to that of untreated control (p-value <0.001 compared to that of untreated control).

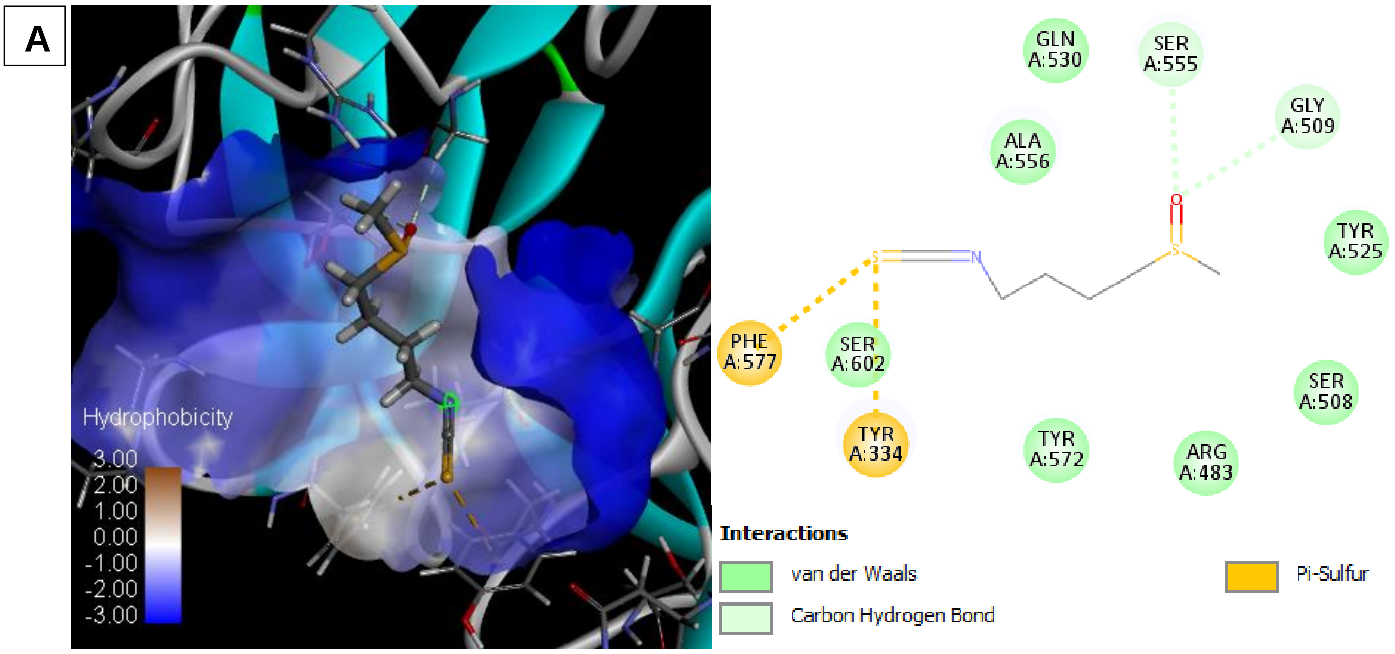

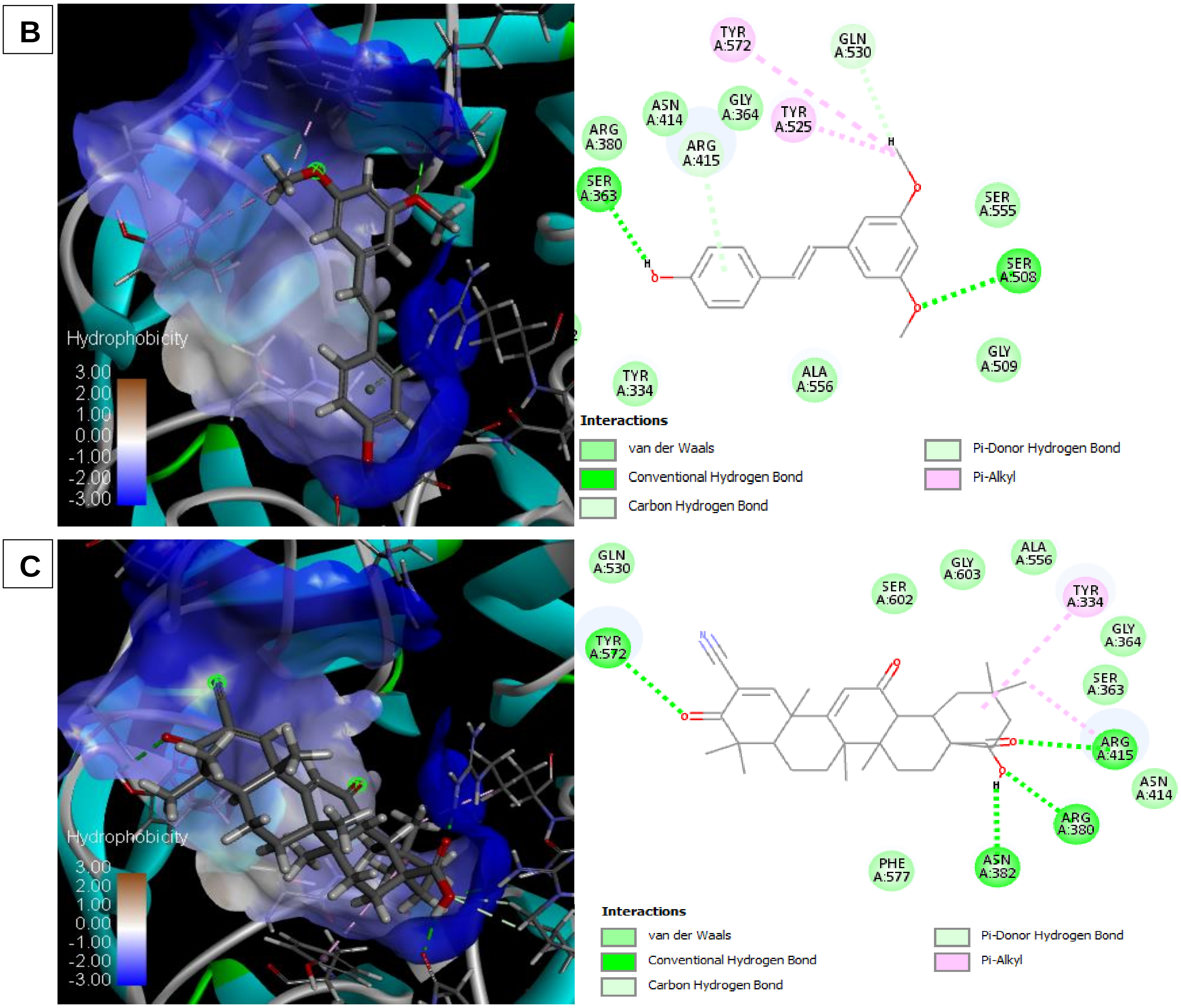

Supplementary Figure S4: Molecular docking of Sulforaphane (A), Pterostilbene (B) and Bardoxolone (C) to Keap1 (4IQK).
